# Bibliometric analysis of gamification in teacher education during the generative AI era: historical trends, present status, and future trajectories

**DOI:** 10.12688/f1000research.178620.1

**Published:** 2026-03-30

**Authors:** Miguel Alberto Rincón Pinzón, Ana Dolores Vargas Sánchez, Leonardo David Glasserman Morales

**Affiliations:** 1 Universidad de La Sabana, Chia, Cundinamarca, Colombia; 2 Universidad Popular del Cesar, Valledupar, Cesar, Colombia; 3 Tecnológico de Monterrey - Campus Ciudad de Mexico, Mexico City, Mexico City, Mexico

**Keywords:** bibliometric analysis, teacher, education, gamification, technology, GenAI, innovation.

## Abstract

Gamification has become a relevant pedagogical approach in teacher education, yet its evolution and relationship with generative artificial intelligence remain fragmented. This study maps the development, intellectual structure, and emerging trajectories of research on gamification in pre-service and in-service teacher education between 2016 and 2025. A bibliometric analysis was conducted on an integrated Scopus and Web of Science dataset. After merging records, removing duplicates, and applying predefined eligibility criteria, 1,223 documents were retained. Performance analysis, co-citation analysis, keyword co-occurrence analysis, and thematic evolution mapping were conducted in Biblioshiny/Bibliometrix, with study identification and selection documented through the PRISMA framework. Scientific output increased markedly over the study period, especially from 2020 onward, and was led mainly by institutions and countries with strong educational innovation capacity. The co-citation network revealed three intellectual foundations: motivational theories of gamification, technology adoption and educational innovation models, and empirical work on game-based learning and serious games. Keyword co-occurrence analysis identified seven thematic clusters organized around gamification, education, artificial intelligence, teacher training, and digital learning. By 2025, the field shows a clear thematic shift toward generative artificial intelligence, adaptive systems, and automated personalization. Research on gamification in teacher education has moved from a focus on motivational design and digital engagement toward more intelligent, data-driven, and adaptive ecosystems. The findings highlight a rapidly expanding field and provide an evidence-based agenda for teacher education, institutional policy, and future studies on the pedagogical and ethical integration of GenAI.

## 1. Introduction

In order to adequately equip educators for the digital era, teacher education is currently experiencing significant transformations. The mechanics of games have emerged as a critical pedagogical instrument for the transformation of learning. Rather than being a trivial trend, gamification enables the motivation of students through playful strategies that captivate their interest and attention. This pedagogical strategy is defined as the incorporation of game elements into non-recreational contexts to enhance learner motivation and engagement. ^1^ Its potential is to stimulate students’ intrinsic motivation. Ryan and Deci ^2^ elucidates this phenomenon by asserting that students’ engagement with learning is substantially enhanced when they experience autonomy, competency, and connection. Gamification is not merely a strategy for motivating students in teacher training. It is a pedagogical approach that enables teachers to experiment, deconstruct, and, in the end, design technology-enhanced learning environments while in training and in practice. ^3, 4^

Teacher education has undergone a transformation and evolution in the context of gamification. Over time, what was initially a literacy system that relied on points, rewards, or external prizes has evolved into a series of more profound learning experiences that are based on the necessity of consciously and systematically utilizing the narrative potential of games, the complexity of their mechanics, authentic challenges, collaborative dynamics, and playfulness. This transformation has enabled gamification to surpass its initial purpose as an incentive and establish itself as a pedagogical strategy that promotes critical reflection, the collective construction of knowledge, and teacher professional development, particularly in educational environments that are increasingly mediated by digital technologies. External motivators, including recognition mechanisms such as rewards and digital badges, were frequently prioritized in early gamified implementations. ^5^ Nevertheless, contemporary methodologies prioritize meaningful gamification, which dynamically connects game mechanics with learning objectives and supports the development of teachers’ professional identity through structured, gamified professional learning experiences. ^6^ Gamified simulations have been identified as innovative instruments for teacher training in recent literature. These virtual environments provide a secure environment for educators to experiment and enhance their pedagogical skills without the risk of real consequences, enabling them to implement classroom management strategies, differentiated instruction, and lesson planning. ^7^

The theoretical basis for this methodology is extensive. From a constructivist standpoint, for instance, knowledge is constructed through a learner’s active engagement with their social context, with each experience contributing to their comprehension of the world. ^8^ Gamified settings are inherently constructivist; they necessitate active involvement, foster experimentation, and facilitate collaboration among participants. Moreover, the framework of Technological Pedagogical Content Knowledge (TPACK) provides a crucial perspective that transcends technical considerations, illustrating how gamification intricately weaves together knowledge, pedagogical approaches, and technological instruments. ^9^ Consequently, educators must grasp how gamification, facilitated by technological tools, can reshape their pedagogical practices and enhance knowledge dissemination within a particular subject domain.

The significant changes in the educational field are reflected in the evolution of gamification in teacher training. The feasibility of this strategy and the perceptions of instructors were the primary focus of early studies. ^10^ In order to investigate the influence of gamification on specific outcomes, including academic performance, engagement, and the development of self-regulated learning skills, subsequent research has examined more rigorous methodological approaches, such as controlled trials and quasi-experimental designs. ^11^ The research field has progressed beyond the mere inquiry of whether gamification is effective. Presently, researchers are intent on comprehending the precise mechanisms, timing, and demographics of its most advantageous efficacy. This transition in research focus has stimulated growing interest in the design of adaptive and personalized gamification systems that align with learners’ individual profiles and preferences. ^12^

In this setting, characterized by the pursuit of increasingly personalized and contextualized learning experiences, artificial intelligence (AI) is beginning to assume a pivotal role in comprehending the trajectory of new advancements in the area. Artificial Intelligence (AI) refers to computer systems that can perceive environmental information, learn from data, and execute tasks that previously necessitated human intelligence, including reasoning, problem-solving, and decision-making. ^13^ In this context, generative artificial intelligence (GenAI) denotes a particular advancement in AI, focused on producing new content (text, images, audio, or code) by analyzing patterns in extensive datasets. This enables progression from analysis to synthesis and creative expression. ^14^ From this viewpoint, large-scale language models (LLMs) like GPT 5.2, Claude 4.5, Gemini 3, LLaMA 4, and Mistral 3.1 have emerged as significant instruments in education, enabling the creation of instructional materials, automated feedback, and the customization of learning experiences.

GenAI is significantly altering the manner in which instruction and learning are conducted in the educational sector. In addition to automating content creation, these technologies provide personalized tutoring through intelligent agents (chatbots) that are capable of supporting the learning process, generating dynamic assessments, and providing immediate and contextualized feedback on student performance. ^15^ Simultaneously, LLMs are beginning to integrate into educational environments that embody the principles of Education 4.0 and even 5.0, ^16^ promoting connected, interactive, and learner-centered learning experiences where technology mediates critical thinking and creativity. ^17^ In addition to their technical capabilities, these tools also ameliorate the administrative burden that has historically restricted the time of teachers, enabling them to concentrate on tasks of greater pedagogical value, such as mentoring, reflection, and deep learning. Nevertheless, this advancement is not without its inherent predicaments, Bozkurt ^18^ arises: What is the impact of this automation on the authenticity of learning and academic integrity? What is the potential for algorithms to replicate or exacerbate cognitive and cultural biases? In the information era, the GenAI is confronted with the challenge of humanizing technology to ensure that it remains a means to education rather than an end in itself, in a context characterized by the digital divide and the redefinition of knowledge.

The convergence of GenAI and gamification presents a transformative horizon for contemporary education, integrating the motivational power of game-based learning with the creative potential of generative models. This synergy addresses the historical challenges in gamified design, particularly in the development of sustainable and dynamic content. GenAI introduces adaptive creativity, which is capable of generating experiences that evolve in accordance with the requirements, interests, and progress of each student, in contrast to traditional systems that relied on fixed narratives and predefined scenarios. It transforms the educational experience into a living, personalized, and emotionally meaningful environment where learning remains fresh, challenging, and pertinent by incorporating contextualized missions, diverse characters, and changing narratives. Rather than supplanting the teacher’s role, this technology functions as a pedagogical and creative ally, enhancing the potential of educators to create more motivating, flexible, and inclusive learning experiences. In this context, its transformative potential is underscored by its ability to encourage active student participation. ^19^

An AI-driven learning platform, for instance, could produce customized case studies for education students, specifically designed to align with their educational stage, subject matter, and particular pedagogical obstacles. Adaptive gamification is significantly enhanced by artificial intelligence. This technology facilitates real-time assessment of interactions, performance metrics, and even the emotional states of both educators and learners. Through sophisticated algorithms, these systems can dynamically modify game mechanics, adjust difficulty levels, and tailor learning pathways, thereby crafting a personalized experience that adapts to the unique requirements and learning tempo of each individual. ^20^ GenAI is advancing the frontiers of educational simulation, especially in the improvement of non-player characters (NPCs). Artificial intelligence-powered educational games provide immersive simulations that revolutionize learning. Students can utilize interactive tools to investigate intricate scenarios with intelligent characters. A prospective educator could rehearse managing challenging classroom scenarios, motivating disinterested students, and obtaining immediate feedback on their pedagogical approaches and empathetic responses. These simulations teach both knowledge and social as well as strategic competencies inside secure and scalable environments. ^21^

GenAI is transforming educational evaluation beyond conventional questionnaires. This system evaluates open interactions in gamified environments, facilitates student decision-making, and produces tailored feedback. It promotes self-regulation and inspires pupils through interactive learning techniques. Examples encompass AI-driven scaffolding systems that promote active engagement ^22^ and metacognitive chatbots facilitate profound introspection. ^23, 24^ Moreover, gamified strategies facilitated by GenAI markedly enhance the quality of pedagogical feedback and formative assessment processes. ^25^ This technical transition represents a fundamental shift rather than a mere increase. AI facilitates a shift from predetermined gamification to emergent gamification, when the learning experience is collaboratively developed between technology and the student. Teacher training entails the creation of contextualized simulations that accurately reflect the complexities and dynamics of a genuine classroom, enabling prospective educators to encounter scenarios akin to actual practice with a degree of realism. Notwithstanding the increasing interest and preliminary advancements in GenAI for teacher training, empirical studies that combine gamification with it are still limited. Research is notably scarce in assessing its influence on pedagogical competences, instructional efficacy, and the learning outcomes of prospective educators. ^26, 27^ Inquiries remain on the potential of AI to augment the cultivation of pedagogical competencies, such as fostering inclusive classrooms or executing novel project-based learning approaches. Thirdly, a critical epistemological conflict emerges: can AI systems tailor learning experiences without constraining teacher autonomy? The difficulty resides in circumventing an algorithmic pedagogy that supplants professional judgment. The fundamental nature of teaching is scrutinized: critical reflection, discernment, and the educator’s personal experience. ^28^

This investigation contributes to the academic discourse by conducting a comprehensive bibliometric analysis of GenAI, teacher training, and gamification. Its objective is to illustrate the intellectual framework and trace the progression of knowledge in this emerging discipline, with a particular emphasis on the influence of GenAI. The analysis is conducted in accordance with the guidelines established by Donthu et al. ^29^ using bibliometric methodologies. This bibliometric study endeavors to address a series of specific research questions by adhering to a precise methodological framework and having a clearly defined objective: RQ1 (Performance): Between 2016 and 2025, what is the quantitative growth trajectory and who are the most influential contributors (authors, institutions, countries, journals) to research on gamification for teacher training? RQ2 (Intellectual basis): Through co-citation analysis, what are the foundational knowledge groups and seminal theoretical pillars that have influenced research on gamification in teacher training? RQ3 (Conceptual structure): How have the main thematic clusters and their interrelationships that define the conceptual structure of the field evolved in the past decade, notably with the recent emergence of GenAI?

## 2. Method

This study uses a quantitative design grounded in bibliometric analysis, which is appropriate for large-scale bibliographic datasets and enables a systematic examination of scientific production, intellectual structure, and emerging trends in gamification for teacher education. In line with Donthu et al., ^29^ the methodological design was organized into four interrelated phases: (1) defining the research scope and objectives, (2) selecting bibliometric techniques and tools, (3) data acquisition and cleaning, and (4) analysis and presentation of findings. To improve transparency in reporting the evidence identification and selection process, the study also applied the PRISMA framework ^30^ to document records identified, screened, excluded, and included, and this process is summarized in a flow diagram. The completed checklist and flow diagram are available in Zenodo as Reporting Guidelines materials. ^61^

### Step 1: Establishing the scope and objectives

The goal is to determine the impact and integration of GenAI, as well as to map the intellectual structure and trace the evolutionary trajectories of knowledge production on gamification in teacher training. Strategically chosen to encompass the recent and rapid emergence of GenAI, which has revolutionized the educational technology landscape, as well as the consolidation of gamification as a mature research topic in education, the research timeframe is defined as the period between January 1, 2016, and November 20, 2025.

### Step 2: Analytical techniques and utilized tools

The study’s established objective is achieved through the use of bibliometric analysis techniques, which are categorized into two groups: performance techniques, which involve the examination of total publications and the number of citations received to identify growth trends and scientific impact in the field; performance by country, which allows for the identification of the primary actors, institutions, and international collaboration networks that influence the research dynamics; and impact techniques, which analyze the performance of the most productive and influential journals. Lastly, an overview of the 10 most cited articles is provided, which reflects the most influential studies and thematic lines that dictate the evolution of knowledge in this field, as well as scientific mapping techniques (keyword co-occurrence analysis and co-citation analysis). In addition to their complementary nature, these methodologies are indispensable for acquiring a thorough understanding of the subject matter. The information is analyzed using Biblioshiny, the interactive web interface of the Bibliometrix utility in R. This interface enables the intuitive interpretation of bibliometric results and the observation of graphs. ^31^ Because this review is bibliometric in nature, conventional effect measures used in intervention syntheses were not applicable; results are therefore presented as bibliometric performance indicators and science mapping outputs.

### Step 3: Data acquisition for bibliometric analysis

**Figure 1.**
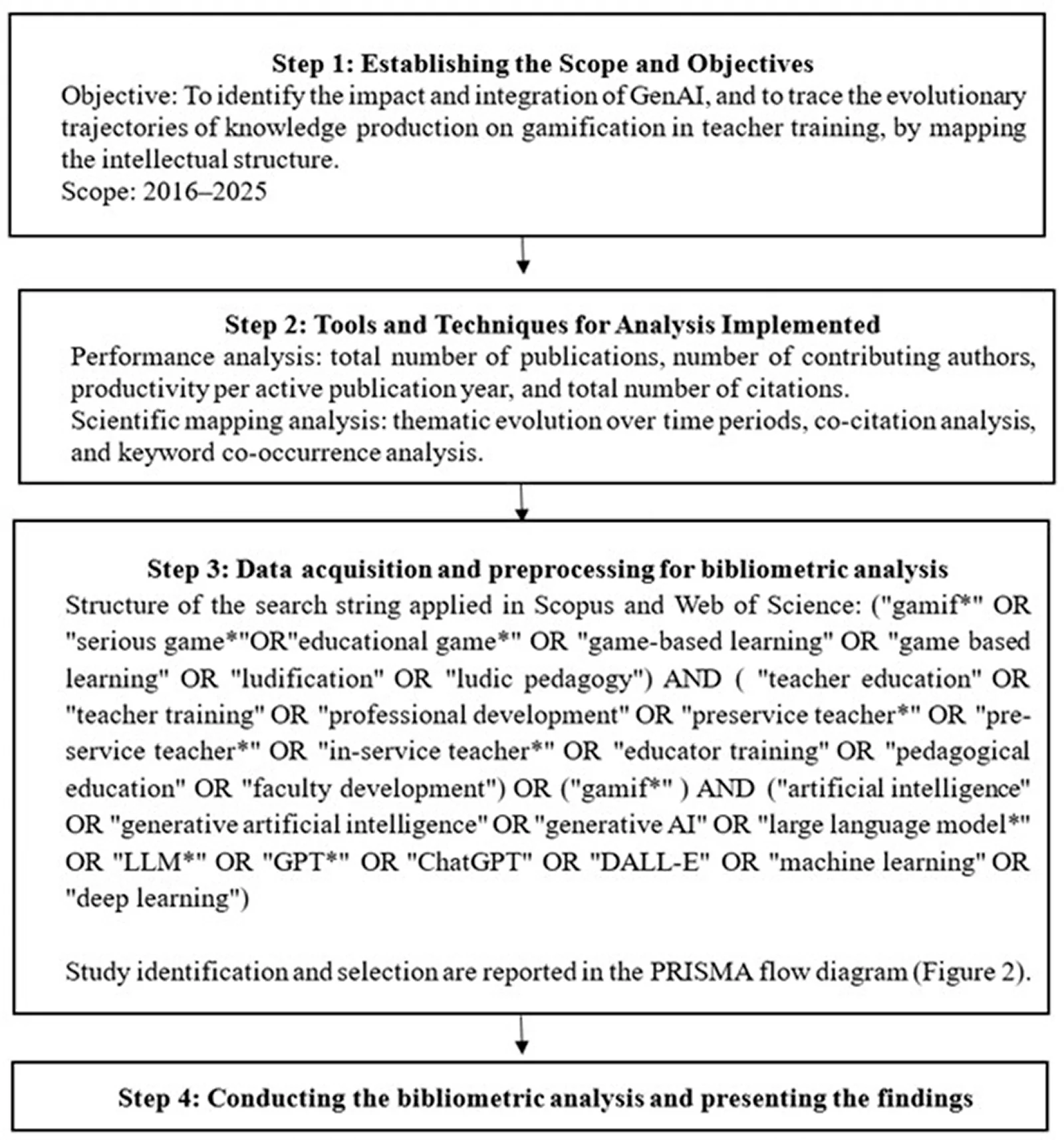
Four-phase bibliometric methodological protocol used in this study, adapted from Donthu et al. (2021).

**Figure 2.**
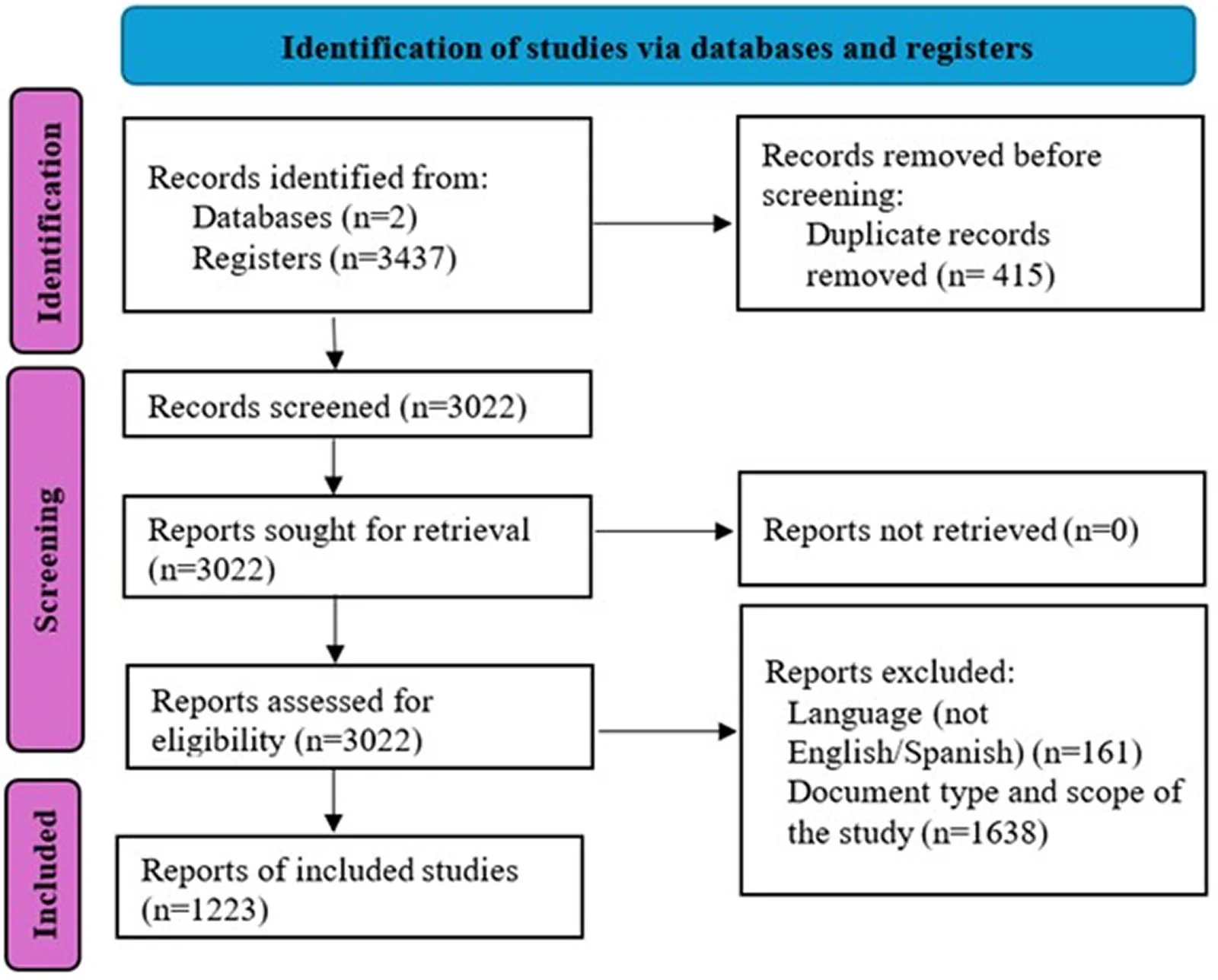
PRISMA flow diagram of the identification, screening, eligibility assessment, and inclusion of records for the bibliometric dataset.

The third step involved data acquisition and preprocessing for the bibliometric analysis, which constituted the empirical basis of the study. Using the predefined search strategy and protocol ( Figure 1), advanced searches were conducted in Scopus and Web of Science to identify records on gamification in teacher education and generative AI. The final database searches were conducted on 20 November 2025. The search retrieved 2,417 records from Scopus and 1,020 records from Web of Science (3,437 records in total). The full identification, screening, eligibility assessment, and inclusion process is reported in the PRISMA flow diagram ( Figure 2).

To construct a unified bibliographic corpus, records exported from both databases were converted into bibliographic data frames and merged in RStudio using the bibliometrix workflow. Duplicate removal was performed during preprocessing with the mergeDbSources function using the argument remove.duplicated = True (combined <− mergeDbSources (wos_data, scopus_data, remove.duplicated = True)), which merges bibliographic data frames and removes duplicated documents identified across common bibliographic fields. In this study, 415 duplicate records were removed at this stage. The function documentation indicates that mergeDbSources merges bibliographic data frames and, when remove.duplicated = True, deletes duplicated documents from the collection.

The deduplicated records were then screened against predefined eligibility criteria in two stages (screening and eligibility). First, one reviewer screened titles/metadata and abstracts, and a second reviewer verified the decisions. Potentially eligible records were then assessed for eligibility using the same criteria: publication period (2016–2025), document type (article or review), language (English or Spanish), and thematic alignment with gamification in teacher education (including records linked to the artificial intelligence and generative AI terms specified in the search protocol). Discrepancies were resolved through discussion and consensus among the authors. No automation tools were used for eligibility decision-making beyond duplicate detection and removal during preprocessing.

Because this study is a bibliometric review focused on mapping scientific production, intellectual structure, and thematic evolution, a formal study-level risk of bias assessment was not performed. The unit of analysis was the bibliographic record and its indexed metadata rather than effect estimates from primary intervention studies; therefore, conventional risk-of-bias appraisal tools were not applicable. Methodological rigor was addressed through predefined eligibility criteria, duplicate removal during preprocessing, and reviewer verification of screening and eligibility decisions.

After duplicate removal and eligibility screening, a final corpus of 1,223 records was retained for bibliometric analysis. The merged and cleaned bibliographic dataset used to construct the final corpus and perform the bibliometric analyses is publicly available in Zenodo. ^60^

### Step 4: Conducting the bibliometric analysis and presenting the findings

The fourth step consisted of conducting the analysis using the bibliometric techniques described in Step 2. The results of this analysis are presented in the following section.

## 3. Results

The database searches identified 3,437 records in total (Scopus: 2,417; Web of Science: 1,020). After merging records and removing 415 duplicates, 3,022 records remained for screening. A total of 1,799 records were excluded during screening/eligibility filtering (non-English/Spanish registers n = 161; non-eligible document types and out of scope articles n = 1,638). The final dataset included 1,223 articles for bibliometric analysis, which came from 657 different sources. These sources were indexed in both Scopus and Web of Science. The data mostly came from the period between 2016 and November 20, 2025, making up 99.8% of the total. The database mostly includes 1,008 research papers and 165 reviews, with a small number of other document categories. The analysis of the data shows a large and dynamic field of study, supported by 50,546 cited references and a group of 4,784 authors, with only 103 working alone. Collaboration is the main way research is done, with an average of 5.07 authors per publication. In addition, international collaboration is common, making up 16.19% of all collaborations. The average age of the documents is only 1.82 years, which confirms that this is a relatively new area of study, characterized by quick expansion and active research.

### 3.1 Analysis of scientific performance

To comprehend the evolution of scientific production regarding gamification in teacher training, it is essential to examine its progression and the contributors who have facilitated this advancement. From this vantage point, scientific performance analysis enables the examination of the quantitative progression of the field and the identification of the authors, institutions, countries, and journals that have substantially contributed to its establishment. According to this purpose, the central inquiry is: What is the trajectory of quantitative growth, and who are the principal contributors (authors, institutions, nations, journals) to research on gamification in teacher training from 2016 to 2025?

***Production growth on an annual basis***

Table 1 illustrates a continuous increase in production from 2016 to 2025. In 2016, 16 publications were discovered, a number that remained modest until 2018 (15–18 records). Beginning in 2019, there was a notable increase (32 articles), which was then amplified throughout the pandemic alongside the enhancement of teaching in hybrid and digital contexts.

**Table 1.** Annual scientific output.

Years	2016	2017	2018	2019	2020	2021	2022	2023	2024	2025
Articles	16	15	18	32	64	94	122	157	267	436

The data indicates that the articles within the corpus garner an average of twelve citations; however, this average conceals a significant disparity. Approximately half of the publications accrue three citations or fewer, a pattern frequently observed in fields experiencing rapid expansion. Conversely, a limited subset of studies significantly surpasses this citation count, with some exceeding four hundred citations. These particular works function as key reference points within the existing literature; they are the most frequently referenced, the catalysts for novel research avenues, and the primary drivers of the topic’s advancement.

***Journals that are more productive and impactful***

Scientific output is disseminated throughout various periodicals, however it is notably concentrated in those that spearhead research in education and technology. The publications encompass Education Sciences (31 articles) and Education and Information Technologies (30), succeeded by Sustainability (25), which have consistently served as platforms for research on gamification and teacher training. Significant are prominent, reputable publications including IEEE Access (21) and Entertainment Computing (20), as well as essential journals in the education sector such as Applied Sciences – Basel (18), Computers & Education (14), and the British Journal of Educational Technology (11). Collectively, these articles illustrate the interdisciplinary character of the discipline and the connections among education, engineering, and computer science. From an impact standpoint, the majority of citations originate from a limited number of journals that dominate the discourse in educational technology. Computers in Human Behavior leads with 671 citations over four articles, while Computers & Education follows with 649 citations in 14 publications. Education Sciences (542 citations), Education and Information Technologies (537), and Interactive Learning Environments (428) are also significant. These periodicals serve as essential platforms for sharing advancements in gamification, educator training, and the application of GenAI in education.

***Authors who have made the greatest impact***

The five most prolific and impactful authors in the studied studies were Gwo-Jen Hwang (six publications), David González-Gómez (five articles), A. Maalel (five articles), P. Washington (five articles), and S. Bennani (four articles). They provide some of the most robust references in the research on gamification, technology-mediated learning, and artificial intelligence within educational environments. Their contributions encompass game-based learning, adaptive gamification, GenAI use, intelligent learning support systems, gamified health applications, and innovative methodologies for designing individualized learning experiences. Table 2 encapsulates their principal contributions and the papers that most effectively exemplify each author’s scholarly trajectory.

**Table 2.** Leading authors in the field.

Author	No. of items	Representative publications
Gwo-Jen Hwang	6	https://doi.org/10.1016/j.compedu.2018.07.001; https://doi.org/10.1111/bjet.13260
David González-Gómez	5	https://doi.org/10.3390/su13031032; https://doi.org/10.1016/j.heliyon.2023.e12795
A. Maalel	5	https://doi.org/10.1002/cae.22477; https://doi.org/10.1007/s42979-024-03491-z
P. Washington	5	https://doi.org/10.2196/26760; https://doi.org/10.2196/49898
S. Bennani	4	https://doi.org/10.1002/cae.22477; https://doi.org/10.1007/s42979-024-03579-6

***Countries and institutions that are at the forefront***

The observed data indicates that knowledge generation is heterogeneous, occurring mostly within a limited number of countries and universities. Spain is the primary reference point, with 171 publications, followed by the United States with 130 and China with 125, suggesting that certain countries contribute far more than others in this domain. This dynamic is evident at institutions such as the University of Granada, the University of Murcia, the University of Barcelona, and the Polytechnic University of Madrid, which spearhead research initiatives and are situated in the nation with the highest production. In the United States, institutions like Pennsylvania State University and the University of Alabama are prominent contributors, reflecting the nation’s second-place rating globally. Despite China’s substantial output of publications, their distribution among various institutions accounts for the absence of any in the Top 10. Countries including Germany, India, the United Kingdom, Australia, Greece, Indonesia, and Italy consistently uphold a significant presence in the domain.

***Articles with the highest citation frequency***

An examination of the most frequently cited texts uncovers a limited yet impactful core of articles that have shaped conceptual and methodological advancements in the discipline. The most influential paper has been cited 454 times, with additional articles cited over 400 and 300 times, primarily focusing on systematic studies about gamification, digital learning, and the applications of artificial intelligence in education. This pattern signifies that the conceptual framework of the domain is founded on a collection of reference books that have influenced theoretical, methodological, and technological choices during the past decade. Table 3 below presents the most cited articles, elucidating the significance of each work within the contemporary research environment.

**Table 3.** Most frequently cited articles in the discipline.

Title	DOI	Citations in total
Technological disruptions in services: Lessons from tourism and hospitality (Buhalis, 2019)	https://doi.org/10.1108/JOSM-12-2018-0398	454
Gamified learning in higher education: A systematic review (Subhash & Cudney, 2018)	https://doi.org/10.1016/j.chb.2018.05.028	440
A novel deep learning method for medical image analysis (Albarqouni et al., 2016)	https://doi.org/10.1109/TMI.2016.2528120	413
Artificial Intelligence in Education: A comprehensive review (2010–2020) (Zhai, 2021)	https://doi.org/10.1155/2021/8812542	398
Gamification in science education: A systematic review (Kalogianakis & Papadakis, 2021)	https://doi.org/10.3390/educsci11010022	287
Teacher support and student motivation in interactive learning environments (Chiu, 2024)	https://doi.org/10.1080/10494820.2023.2172044	256
Do AI chatbots improve students’ learning outcomes? (Wu, 2024)	https://doi.org/10.1111/bjet.13334	216
A scoping review of digital game-based learning for ASD (Hung, 2018)	https://doi.org/10.1016/j.compedu.2018.07.001	191
Learning analytics and gamification in higher education (Ng, 2023)	https://doi.org/10.1007/s10639-022-11491-w	164
Effects of game-based learning on engagement and retention (Goksun, 2019)	https://doi.org/10.1016/j.compedu.2019.02.015	161

### 3.2 Scientific mapping analysis

Discern the concepts, theories, and scientific groups that have influenced its evolution over time. Co-citation analysis serves as a valuable instrument for identifying the knowledge groups that have impacted studies on gamification in teacher training. This section’s guiding question aims to elucidate the organization and interrelation of the works and theoretical frameworks that have underpinned the field’s development. From this viewpoint, the guiding inquiry RQ2 (intellectual foundation) is: What are the fundamental knowledge clusters and pivotal theoretical frameworks that have influenced research on gamification in teacher training, as indicated by co-citation analysis?

***Author co-citation network***

**Figure 3.**
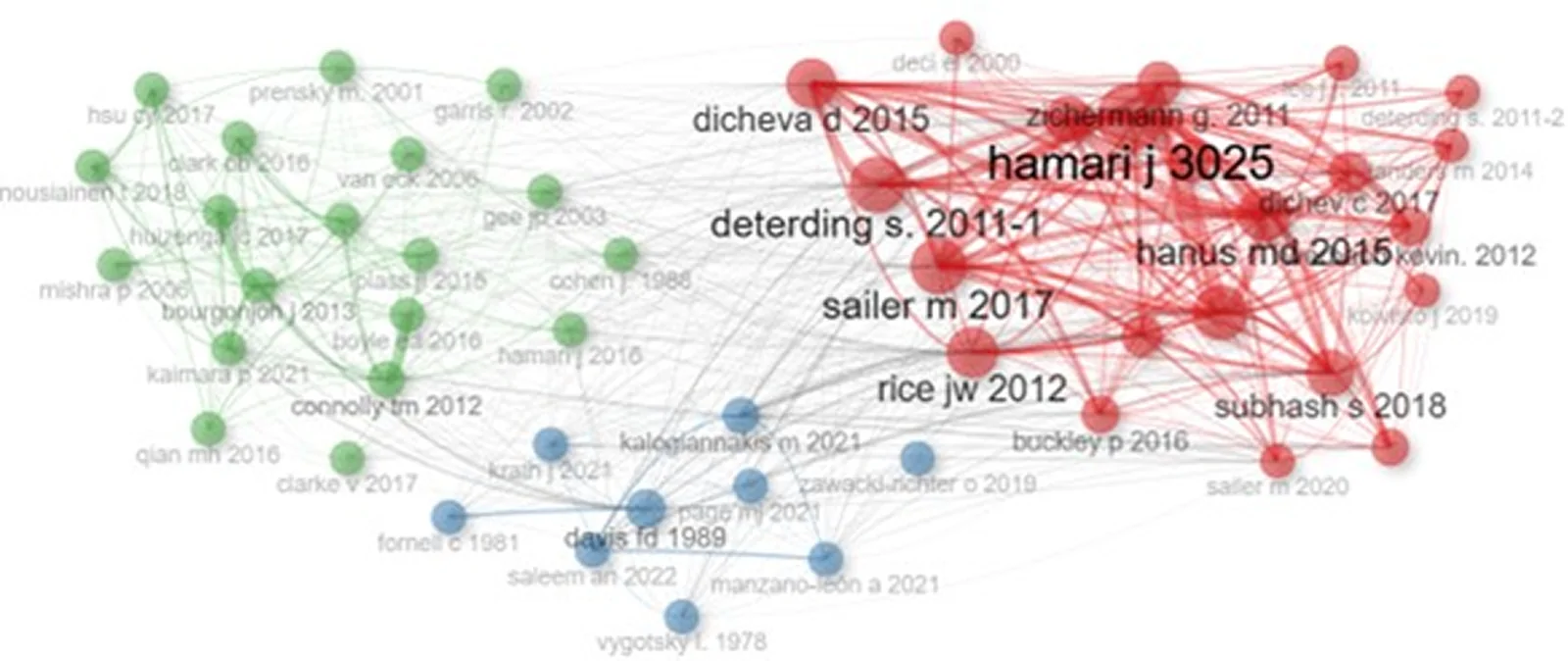
Illustrates the intellectual structure of the field and the co-citation network of references.

The co-citation analysis demonstrates a highly consolidated intellectual structure, which is composed of three thematic clusters that articulate the theoretical, methodological, and empirical underpinnings of current research in educational artificial intelligence, game-based learning, and gamification. The co-citation network was generated by assigning a minimum co-citation threshold that was equivalent to the software’s default value (1 occurrence). This approach enabled the identification of all structural relationships between the references cited in the corpus and the Louvain community detection algorithm, resulting in three well-defined clusters. The co-citation network generated from the set of references is illustrated in Figure 3, which plainly demonstrates the three clusters that form the intellectual foundation of the field.

The works that have established the conceptual language of gamification and its psychological foundations are gathered in Cluster 1 - Fundamentals of gamification and motivation (red). The principles that govern motivational design and the use of game elements are consolidated in contemporary syntheses that systematize instructional design approaches in gamification and map how gamified learning is conceptually framed and operationalized in educational settings. ^32, 33^ In addition to these, empirical studies and evaluations that assess its educational efficacy ^34– 36^ are present, as well as traditional theoretical frameworks on intrinsic and extrinsic motivation. ^2^ The conceptual core of the discipline is comprised of this cluster, which elucidates the nature of gamification, its design, and the psychological principles under which it operates in educational settings.

Cluster 2 - Technology adoption, teaching innovation, and educational AI (blue) comprises references that elucidate the pedagogical foundations that facilitate the integration of digital tools and artificial intelligence in education, as well as the processes of technology adoption. It encompasses contributions such as the Technology Acceptance Model (TAM), which identifies the cognitive determinants that influence the adoption of computer systems that support digital literacy, ^37^ reviews on AI applied to education, ^38^ and studies that concentrate on digital competencies and teaching innovation. The most consistent work within the cluster is that of Kalogiannakis et al. ^39^ providing an explanation of the dynamics through which instructors and students integrate technologies, such as AI and gamified systems, into their educational practices, while also projecting the methodological and institutional foundation of the field.

Cluster 3 - Game-based learning, serious games, and empirical evidence (green) consolidates empirical and experimental contributions that affect the educational efficacy of digital and serious games, delineating the cognitive, motivational, and behavioral outcomes anticipated from game-based learning. ^40– 42^ It offers a theoretical framework for comprehending the principles of building learning-focused fun experiences, incorporating cognitive, emotional, and motivational models. ^43^ The cluster additionally incorporates following empirical investigations that analyze phenomena in conjunction with research on contextual learning, storytelling, and interactive experience design. This study examines the influence of engagement on academic achievement, ^44^ assesses the perception and implementation of digital games in secondary education, ^45^ and explores the pedagogical strategies required for the integration of games into teaching practices. ^46^ This delineates the empirical and applied foundation of the discipline, consistently demonstrating the efficacy of various game-based tactics, the conditions under which they operate, and their impact on student motivation, performance, and cognitive processes.

**Conceptual framework and thematic development**

In addition to theoretical foundations, it is crucial to comprehend the organization of academic discourse on gamification in teacher training and its evolution over time. Examining topic clusters and their temporal history reveals the maturation of the discipline and identifies emerging research trajectories. The primary inquiry of this section, RQ3 (conceptual structure), is: What are the principal thematic clusters and their interconnections that delineate the conceptual framework of the field, and how have these themes developed over the past decade, especially in light of the recent emergence of GenIA?

***Keyword co-occurrence analysis***

The unified field KW_Merged, which integrates Author Keywords, was employed to guarantee semantic consistency. Association Strength, a technique that is recommended for the identification of robust relational patterns between terms, was employed to normalize the network. A minimum threshold of five occurrences was established to ensure that only concepts with structural significance in the field were retained, which were grouped into seven thematic clusters. The network was reviewed for overall frequency ( Table 4), which revealed that the most frequently occurring terms were: gamification, artificial intelligence, education, game-based learning, learning, machine learning, higher education, motivation, serious games, and teacher training. These frequencies suggest that recent research is primarily focused on the intersection of artificial intelligence, education, and gamification.

**Table 4.** Predominant keywords in the co-occurrence analysis.

Word	Frequencies
Gamification	416
Artificial intelligence	168
Education	99
Game-based Learning	90
Learning	78
Machine learning	73
Higher education	71
Motivation	52
Serious games	50
Teacher training	45

**Figure 4.**
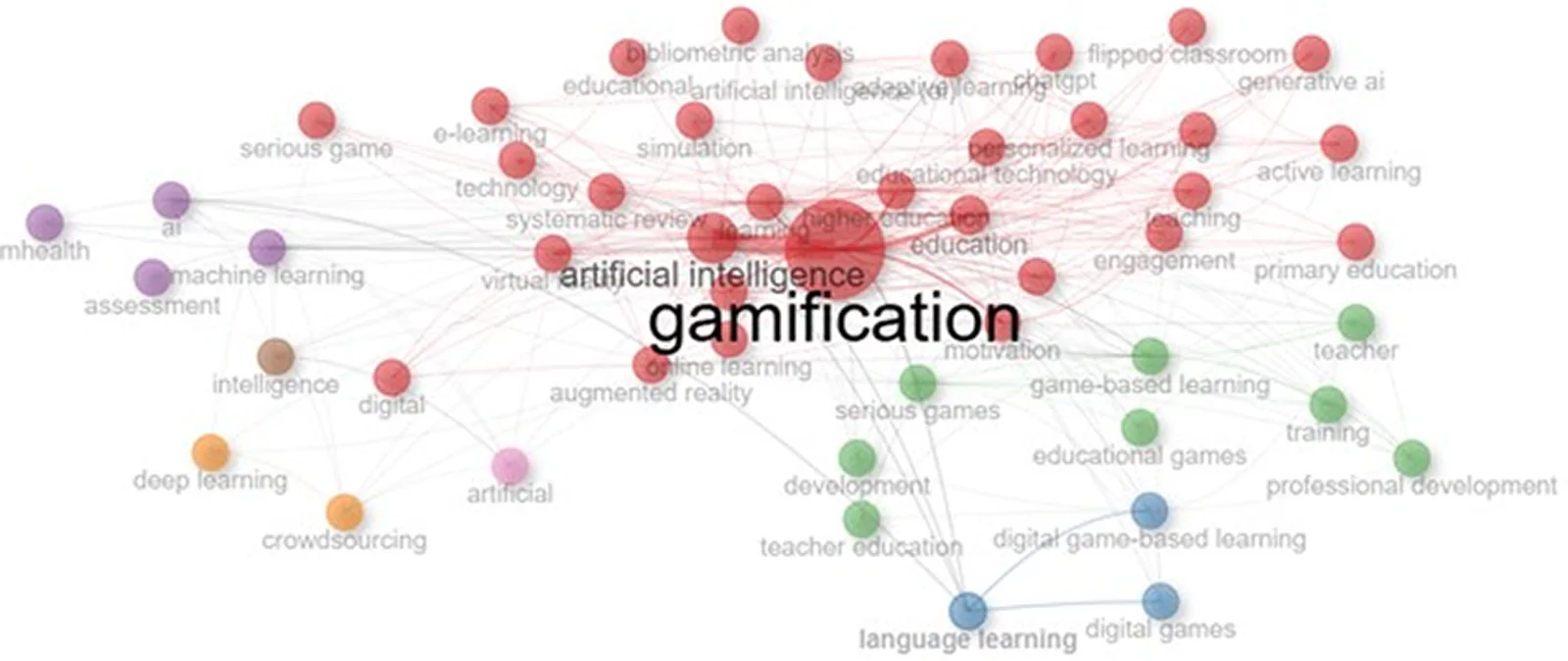
Conceptual framework of the domain.

Figure 4 illustrates the co-occurrence network constructed by the Louvain algorithm, identifying seven thematic clusters denoted by colors, which elucidate the conceptual organization of the field.

The structural center of the conceptual map is represented in red by Cluster 1, which is the central core of gamification, AI, and education. It consolidates frequently used terms, including gamification, artificial intelligence, education, learning, higher education, motivation, and teacher training, with emerging concepts such as ChatGPT, generative AI, personalized learning, augmented reality, and virtual reality. This node’s density indicates that gamification has become a cross-cutting approach, with pedagogical objectives associated with motivation, student engagement, active learning, and higher education, as well as associated with emergent technologies. In this regard, the cluster functions as the conceptual matrix of the field, from which the other lines of research are structured and connected.

Cluster 2 – Digital Learning and Educational Games – encompasses concepts such as *digital game-based learning, digital games, and language acquisition.* Depicted in blue, it emphasizes a particular trend about the creation and utilization of digital games as educational instruments. This theme area seeks to assess the extent to which game-based digital environments enhance cognitive, linguistic, and disciplinary competencies via interactive activities. The arrangement indicates that learning facilitated by digital games is a well-established subfield with clearly defined educational applications.

Cluster 3 – Teacher Training and Professional Development, denoted by the color green, encompasses terminology related to *teacher training, including game-based learning, serious games, educator, teacher education, training, and professional development.* The convergence of these concepts illustrates an increasing interest in comprehending how game-based experiences and gamified dynamics might enhance instructional skills in both pre-service and working educators. Consequently, the cluster serves as a pivotal element in the domain, linking gamification with authentic educational practices.

Cluster 4 – AI, Machine Learning, and Assessment, denoted in yellow, encompasses concepts such as machine *learning, AI, assessment, and mHealth,* illustrating the computational and analytical aspect of the concept map. This area includes research on prediction models, automated evaluation, intelligent systems, and learning analytics. Its contribution is essential for comprehending the integration of AI and machine learning into gamified experiences, particularly for feedback mechanisms, personalization, and the monitoring of academic success.

Cluster 5 – Deep Learning and Collaborative Participation, denoted in orange and consisting of *deep learning and crowdsourcing*, is limited in quantity yet adds substantial depth to the theme network design. This compiles research on sophisticated technology for deep neural networks and strategies for collaborative engagement that facilitate intelligent systems in education. The peripheral location, coupled with its connectivity to other clusters, indicates that this field is emerging as a significant complement to initiatives that integrate artificial intelligence and education.

Cluster 6 – Intelligence, depicted in brown, serves as a cognitive and computational construct, encompassing the term *intelligence* as a cross-disciplinary category that integrates viewpoints from cognitive psychology and intelligent systems. Its function inside the network serves as a conceptual axis linking research on reasoning, cognitive abilities, and computational models seen in gamified educational settings.

Cluster 7, which is depicted in black and is characterized by the term “artificial,” functions as a technical node that is associated with the development, infrastructure, and concepts of artificial technologies and their connection to AI and gamification. Despite its diminutive size, it enables the articulation of network segments that link technological discussions with pedagogical applications.

***Periodical progression of themes***

Thematic evolution enables us to observe the evolution of research lines in gamification and teacher training over the analyzed period. Two periods were generated to organize themes according to their co-occurrence and conceptual continuity, as the database encompasses publications from 2016 to 2025: 2016–2024 and 2025.

*Period 1 (2016–2024): Conceptual consolidation and technical proliferation*

**Figure 5.**
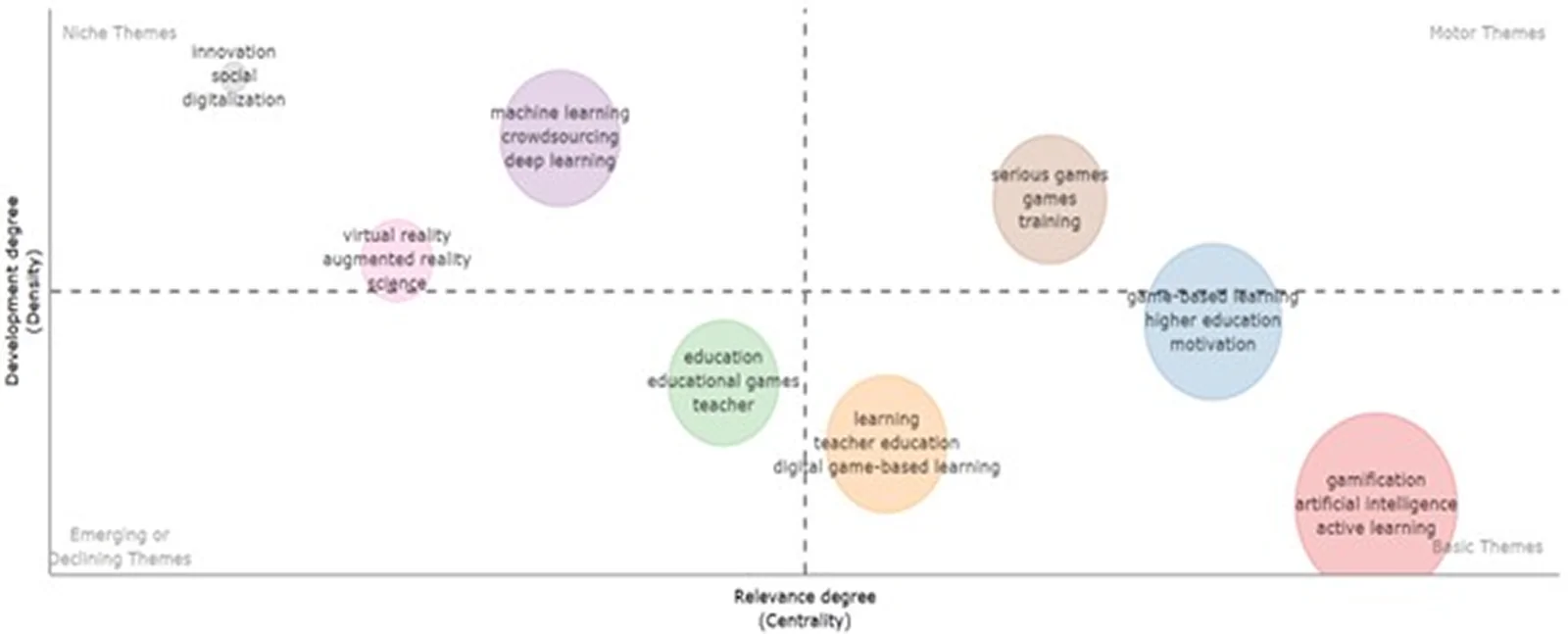
Period 1 thematic map (2016–2024).

In this initial phase (2016–2024), the theme framework indicates that gamification serves as the pivotal element of the domain, linking to learning, higher education, motivation, active learning, and educational technology. This structure illustrates that the research concentrated on establishing a robust conceptual framework, wherein motivation, active learning, and digital interaction served as the foundational pillars directing academic output. Concurrently, game-based learning and serious games emerge as well-established theme areas, providing empirical-applied evidence to the subject, whereas concepts such as machine learning, virtual reality, and augmented reality manifest as burgeoning technical domains. This convergence of pedagogy, motivation, and technology indicates a time of conceptual consolidation in the field, wherein experimental and computational methodologies are increasingly integrated into gamified activities. Figure 5 depicts the theme map of Period 1, highlighting the centrality of gamification and the peripheral distribution of developing technologies.

*Period 2 (2025): Emergence of disruptive challenges related to GenAI*

**Figure 6.**
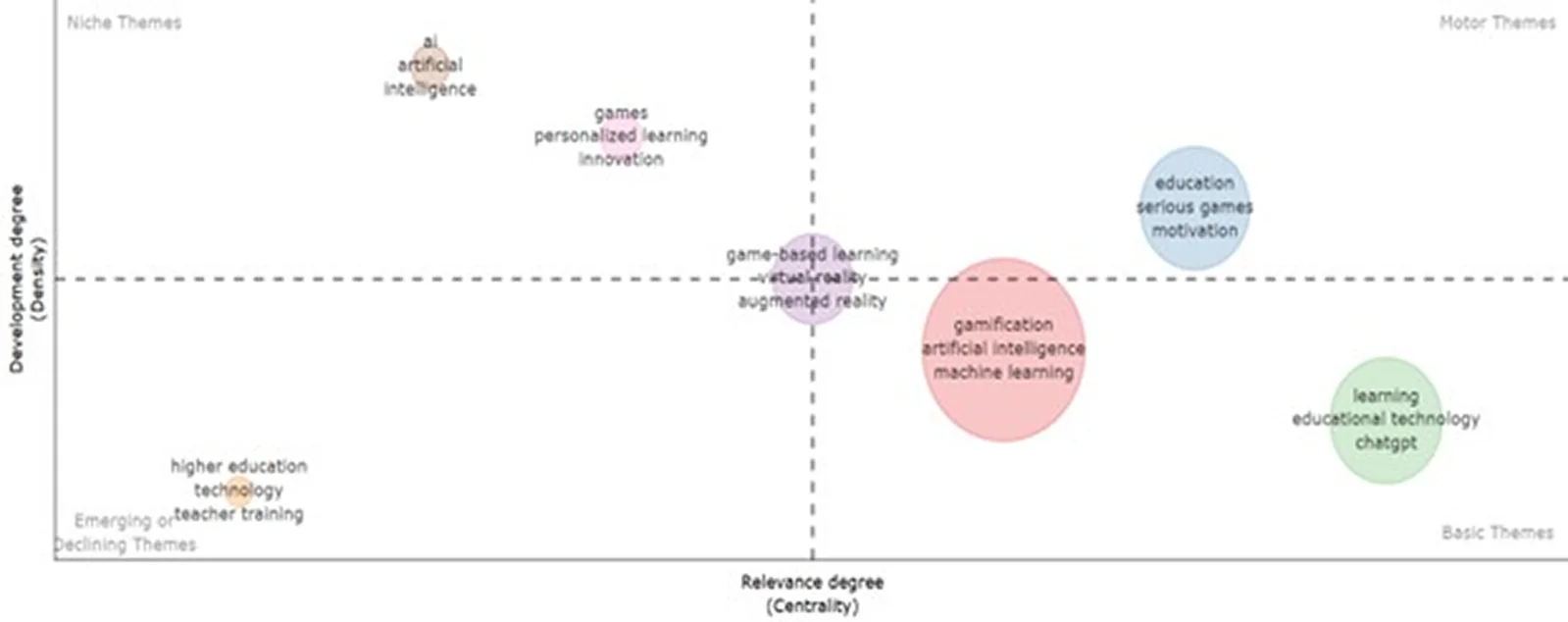
Thematic map of Period 2 (2025).

Thematic evolution exhibits a substantial transition toward concepts associated with generative artificial intelligence during the second period (2025). The field’s orientation toward automation models, intelligent personalization, and automated content generation is indicated by the emergence of new core terms such as artificial, interactive, and technology, which directly integrate with the traditional axes of gamification, education, and learning. This period is characterized by a period of methodological transition, during which chatbots, adaptive systems, and GenAI-based platforms become increasingly prevalent. Game-based learning continues to be a significant thematic thread, despite this, as it enables game dynamics to remain fundamental, albeit in more intricate technological ecosystems. This transition is illustrated in Figure 6, which depicts the emergence of AI-centric thematic nodes and the reconfiguration of centrality between 2025 and the previous period.

## 4. Discussion

This bibliometric study was designed to map the intellectual structure and evolutionary trajectories of research on gamification in teacher training, with a particular emphasis on the emergent impact of GenAI. The findings indicate that a small number of countries and institutions are responsible for the majority of scientific output, and that concepts such as gamification, artificial intelligence, and higher education are central to academic production. This enables universities with established capacities in pedagogical innovation and data analysis to be the primary driving force behind advancements in the field. ^47^ Recent research has demonstrated that numerous universities have integrated digital games, simulations, and gamified activities into teacher training and the cultivation of transversal skills, which is consistent with this trend. ^48^ Research indicates that the utilization of learning games fosters the perception of enhanced soft skills and fosters favorable attitudes toward these methodologies. ^49^ Conversely, business simulation games have been observed to assist students in the application of theoretical content in decision-making contexts that are more closely related to professional practice. ^50^

This thematic expansion is consistent with the intellectual clusters that were identified in the co-citation network. The cluster that concentrates on game-based learning and serious games exhibits a distinct shift from motivational approaches to instructional design proposals that are designed to produce quantifiable effects on academic performance, professional skills development, and employability. This interpretation is supported by recent empirical evidence: serious gaming programs for graduates have been discovered to enhance the perception of employability and the development of transferable skills, ^49^ and the incorporation of management simulations like FLIGBY has been demonstrated to facilitate the development of leadership and decision-making skills in university settings. ^51^ These discoveries elucidate the reason why thematic maps frequently feature topics related to game-based learning as drivers of the field. These topics provide tangible evidence of the influence of gamified strategies on professional performance and learning. The bibliometric results for teacher training demonstrate a cluster that is clearly differentiated and organized around teacher training, teacher education, training, and professional development. This pattern is consistent with empirical evidence that suggests that future teachers are beginning to acknowledge digital games as legitimate classroom tools, despite their continued expression of apprehensions regarding the necessity of curricular coherence, the increased burden, and the challenges associated with assessment. ^52^

A key focus of the conversation is the growing significance of artificial intelligence, particularly GenAI technologies. Co-occurrence analysis indicates that terms like artificial intelligence, machine learning, generative AI, and ChatGPT are intricately associated with gamification and education, implying that the contemporary discourse has evolved beyond conventional game mechanics to include adaptive systems, learning analytics, and conversational agents. Recent empirical literature unequivocally endorses this convergence: AI-related digital competencies significantly affect teachers’ integration of smart technologies into their daily practices, ^53^ whereas perceptions of utility and self-efficacy are critical determinants of pre-service teachers’ readiness to adopt AI tools in educational environments. ^54^ University faculty acknowledge ChatGPT’s capacity to offer formative feedback and assist in academic writing, while concurrently voicing ethical apprehensions regarding plagiarism, technological reliance, and algorithmic biases. ^55^ The thematic transition from Period 1 (2016–2024) to Period 2 (2025) is notable, characterized by a shift from an emphasis on conceptual consolidation and technological advancement to the rise of disruptive themes linked to GenAI, which is increasingly reshaping educational methodologies. ^18^

The integration of generative artificial intelligence (GenAI) enhances adaptive and intelligent gamification, as it enables real-time modifications to learning experiences based on user performance, needs, and behavior, in contrast to traditional systems that rely on fixed pathways. Current data substantiates this advancement, illustrating that generative models can dynamically modify material and learning sequences, thereby providing individualized settings that enhance student engagement and learning outcomes. ^56^ Moreover, research indicates that educational platforms including GenAI systematically modify obstacles and resources, hence enhancing engagement and promoting more effective learning pathways in comparison to traditional systems. ^57^ Moreover, GenAI’s capacity to autonomously generate resources, storylines, and situations is addressing a significant obstacle to the extensive implementation of gamified strategies: the time and effort necessary to develop high-quality educational materials. Consequently, it is clear that the amalgamation of game mechanics with customization algorithms facilitates the development of activities and challenges customized to the user’s profile, thereby augmenting both motivation and educational significance. ^58^ Likewise, the integration of GenAI tools with gamified methodologies has demonstrated favorable outcomes in student motivation and engagement by providing more contextualized experiences that adapt to user performance. ^59^

This bibliometric review should be interpreted considering limitations of both the evidence base and the review process. At the evidence level, the synthesis relies on indexed bibliographic metadata (titles, abstracts, keywords, citations, and reference lists), which may not capture implementation details of gamified teacher-education interventions (e.g., mechanics, pedagogical design, context, and assessment) and is influenced by citation time-lags and visibility effects that can understate the impact of more recent studies. At the process level, the search was restricted to Scopus and Web of Science and to publications from 2016–2025, document types limited to articles/reviews, and English/Spanish language outputs; consequently, relevant studies outside these databases, formats, or languages may be underrepresented. In addition, retrieval depends on database indexing practices and query syntax, and although screening/eligibility decisions were verified and resolved by consensus and preprocessing included standardized merging and deduplication, some misclassification and metadata inconsistencies cannot be fully excluded; likewise, conventional risk-of-bias and certainty assessments were not applicable given the bibliometric (non-effect-size) nature of the synthesis. Despite these constraints, the results provide actionable implications: for practice, teacher-education programs can use the identified thematic clusters and emerging GenAI-related trajectories to prioritize professional development, instructional design, and ethical critical use of AI-enabled tools; for policy, institutions and education authorities may support evidence-informed guidelines for GenAI integration, capacity-building, and responsible innovation aligned with data protection and academic integrity; and for future research, the mapped gaps suggest the need for more context rich empirical studies, comparative designs across regions and teacher-education settings, inclusion of additional databases grey literature, and mixed-method approaches that connect bibliometric trends with implementation quality and learning outcomes.

## 5. Conclusions

The data indicates a consistent development trajectory, which has recently intensified, in relation to the first question (RQ1, performance), as indicated by the questions that guided this study. This field is youthful, dynamic, and expanding at a rapid pace, as evidenced by the scientific output produced between 2016 and 2025. This progress is primarily concentrated in a small number of countries, universities, and journals, with a significant number of university institutions that have dedicated themselves to educational innovation and learning analytics. In an ecosystem where highly influential works coexist with a diverse base of exploratory studies, the combination of high collaboration rates, citation concentration in a small core of articles, and a diversity of journals is indicated.

The second question (RQ2, intellectual basis) indicates that the intellectual framework of the field is founded on three key components: the motivational underpinnings of gamification, frameworks for technology adoption and educational innovation, and empirical data from game-based learning and serious games. The co-citation analysis indicates that contributions on motivation, game element design, and relevant psychological theories are not standalone references; instead, they collectively form a cohesive framework that has established the field’s terminology, concepts, and design standards. Consequently, research on technology adoption and educational AI establishes a framework for comprehending the integration of these tools into pedagogical practices, while empirical investigations of games and simulations elucidate their effectiveness, target demographics, and contextual conditions.

The most intriguing discovery of the study is revealed in the third question (RQ3, conceptual structure and thematic evolution): the field is reconfiguring itself in response to the convergence of artificial intelligence, higher education, and gamification. The seven keyword clusters that have been identified demonstrate a highly stable core that is concentrated on gamification, artificial intelligence, and education, as well as specific areas that are dedicated to digital games, teacher training, machine learning, and advanced technologies. This transition is plainly demonstrated by comparing the period from 2016–2024 to 2025. It transitions from a phase of technological expansion and conceptual consolidation, which was characterized by traditional educational technologies and active learning, to a period in which terms associated with generative artificial intelligence, automated personalization, and intelligent interactive environments emerge. In other words, the thematic map suggests that the inquiry is no longer merely about how to gamify teacher training; rather, it is about how to do so in collaboration with intelligent systems that are capable of adjusting content, challenges, and feedback in real time.

## F1000 AI policy

In accordance with the F1000Research AI Policy, ChatGPT-5.2 (OpenAI) was used for language and style revision of selected sections of the article in order to improve clarity, grammar, and readability in English. The tool was not used to generate original scientific content, make methodological decisions, conduct the bibliometric analysis, interpret findings, or create or manipulate figures, tables, formulas, or underlying data. All AI-assisted outputs were critically reviewed, edited, and validated by the authors, who take full responsibility for the originality, accuracy, and integrity of the final manuscript.

## Data Availability

Rincón Pinzón, M. A., Vargas Sánchez, A. D., & Glasserman Morales, L. D. (2026). Bibliometric analysis of gamification in teacher education during the generative AI era historical trends, present status, and future trajectories [Data set]. Zenodo. https://doi.org/10.5281/zenodo.18773178 Rincón Pinzón MA, Vargas Sánchez AD, Glasserman Morales LD. Reporting guidelines materials (PRISMA 2020): checklist and flow diagram for bibliometric analysis of gamification in teacher education during the generative AI era: historical trends, present status, and future trajectories. Zenodo; 2026. https://doi.org/10.5281/zenodo.18779136
