# Supplementary material for: Bibliometric analysis of gamification in teacher education during the generative AI era: historical trends, present status, and future trajectories
Source: F1000Res. 2026 Jun 23;15:455. Originally published 2026 Mar 30. [Version 2] doi: 10.12688/f1000research.178620.2 (PMC13550912; doi:10.12688/f1000research.178620.2)
Supplement: Peer review history

# Reviewer report: Reviewer response for version 2

**DOI:** 10.5256/f1000research.203399.r498895

**Published:** 2026-07-27

**Authors:** Rajiansyah Rajiansyah

**Affiliations:** 1 Universitas Mulawarman, Samarinda, East Kalimantan, Indonesia

The manuscript presents several clustering analyses based on bibliometric mapping. While the visualizations are informative, the methodology would be strengthened by briefly discussing the robustness of the identified clusters. For example, the authors could explain why the selected clustering algorithm (e.g., the Louvain algorithm) was chosen, describe any parameter settings used during clustering, or acknowledge that formal validation or sensitivity analysis was not performed. Including this discussion would improve the transparency and credibility of the bibliometric analysis, although it does not undermine the overall findings of the study. Overall, this manuscript presents a valuable study that makes a meaningful contribution to the field. It addresses a relevant topic, applies an appropriate and systematic methodology, and provides useful insights that can support future research and practice in educational technology. The manuscript is generally well organized, and the figures, tables, and references effectively support the presented findings. I identified a few minor issues related to the accuracy of terminology and the clarity of methodological descriptions. Addressing these comments would further improve the quality, precision, and transparency of the manuscript. Subject to these minor revisions, I believe the article is suitable for publication.

Are the rationale for, and objectives of, the Systematic Review clearly stated?

Yes

Is the statistical analysis and its interpretation appropriate?

Yes

If this is a Living Systematic Review, is the ‘living’ method appropriate and is the search schedule clearly defined and justified? (‘Living Systematic Review’ or a variation of this term should be included in the title.)

Yes

Are sufficient details of the methods and analysis provided to allow replication by others?

Partly

Are the conclusions drawn adequately supported by the results presented in the review?

Yes

Reviewer Expertise:

Gamification, Game Theory, Decision Theory, and Programming

I confirm that I have read this submission and believe that I have an appropriate level of expertise to confirm that it is of an acceptable scientific standard.

# Reviewer report: Reviewer response for version 2

**DOI:** 10.5256/f1000research.203399.r498899

**Published:** 2026-07-10

**Authors:** Febblina Daryanes

**Affiliations:** 1 Universitas Riau, Pekanbaru, Riau, Indonesia

To enhance transparency and reproducibility, I recommend including the full search queries (including Boolean operators, truncation symbols, field tags, and any database-specific filters) either in the main manuscript or as supplementary material. This would allow future researchers to replicate the study more accurately.

 A brief discussion explaining the rationale for selecting only Scopus and Web of Science would strengthen the methodological rigor.

 Why was the period from 2016 to 2025 selected for this bibliometric analysis? Please provide a clear justification for the chosen time span and explain why earlier publications were excluded from the analysis.

 The manuscript presents several clustering analyses; however, no information is provided regarding the stability or validation of the identified clusters. It would strengthen the study if the authors discussed the robustness of the clustering results or acknowledged this as a methodological limitation. Even a brief explanation regarding the chosen clustering algorithm and its implications would improve the credibility of the findings.

 The discussion regarding the impact of Generative AI appears to be somewhat overstated and should be interpreted with greater caution. Several statements (e.g., suggesting that the field has been "fundamentally restructured" by Generative AI) are not fully supported by the bibliometric evidence presented. Based on the results, Generative AI-related keywords only emerged prominently toward the end of the study period, particularly around 2025. Therefore, the current findings primarily indicate an emerging research trend rather than a fundamental transformation of the field. Since bibliometric analysis reveals publication patterns and thematic evolution, it cannot by itself substantiate claims of an epistemological or structural shift within the discipline. I encourage the authors to moderate these claims and ensure that the discussion remains closely aligned with the evidence provided by the bibliometric analysis.

Are the rationale for, and objectives of, the Systematic Review clearly stated?

Yes

Is the statistical analysis and its interpretation appropriate?

Yes

If this is a Living Systematic Review, is the ‘living’ method appropriate and is the search schedule clearly defined and justified? (‘Living Systematic Review’ or a variation of this term should be included in the title.)

Not applicable

Are sufficient details of the methods and analysis provided to allow replication by others?

Partly

Are the conclusions drawn adequately supported by the results presented in the review?

Partly

Reviewer Expertise:

Biology education; education

I confirm that I have read this submission and believe that I have an appropriate level of expertise to confirm that it is of an acceptable scientific standard, however I have significant reservations, as outlined above.

# Reviewer report: Reviewer response for version 2

**DOI:** 10.5256/f1000research.203399.r496044

**Published:** 2026-07-08

**Authors:** Louis Robert C. Sison

**Affiliations:** 1 Bulacan State University, Bulacan, Philippines

I have no further comments to make. The identified comments were addressed by the authors.

Are the rationale for, and objectives of, the Systematic Review clearly stated?

Yes

Is the statistical analysis and its interpretation appropriate?

Yes

If this is a Living Systematic Review, is the ‘living’ method appropriate and is the search schedule clearly defined and justified? (‘Living Systematic Review’ or a variation of this term should be included in the title.)

Not applicable

Are sufficient details of the methods and analysis provided to allow replication by others?

Yes

Are the conclusions drawn adequately supported by the results presented in the review?

Yes

Reviewer Expertise:

Mathematics Education, Pre-service Teacher Education

I confirm that I have read this submission and believe that I have an appropriate level of expertise to confirm that it is of an acceptable scientific standard.

# Reviewer report: Reviewer response for version 2

**DOI:** 10.5256/f1000research.203399.r496507

**Published:** 2026-07-08

**Authors:** Fei Ping Por

**Affiliations:** 1 Tunku Abdul Rahman University of Management and Technology, Kuala Lumpur, Federal Territory of Kuala Lumpur, Malaysia

This paper addresses a worthwhile topic and presents a substantial volume of bibliometric data. The four-period evolutionary model and the documentation of GenAI's emerging presence are genuine contributions. The PRISMA-guided selection process and the use of Biblioshiny/Bibliometrix lend methodological credibility, and the public availability of the dataset on Zenodo is commendable for reproducibility.

 The manuscript is well-written, and no further revision is required from me.

Are the rationale for, and objectives of, the Systematic Review clearly stated?

Yes

Is the statistical analysis and its interpretation appropriate?

Yes

If this is a Living Systematic Review, is the ‘living’ method appropriate and is the search schedule clearly defined and justified? (‘Living Systematic Review’ or a variation of this term should be included in the title.)

No

Are sufficient details of the methods and analysis provided to allow replication by others?

Yes

Are the conclusions drawn adequately supported by the results presented in the review?

Yes

Reviewer Expertise:

English Language Studies, Educational Management, Educational Technology

I confirm that I have read this submission and believe that I have an appropriate level of expertise to confirm that it is of an acceptable scientific standard.

# Reviewer report: Reviewer response for version 1

**DOI:** 10.5256/f1000research.197029.r481082

**Published:** 2026-05-18

**Authors:** Louis Robert C. Sison

**Affiliations:** 1 Bulacan State University, Bulacan, Philippines

1. The abstract captures the essence of the entire paper, which includes the purpose, methods, results, and conclusion. 

 2. Include keywords that are not explicitly included in the title for better networking. 

 3. For the introduction (Paragraph 9), strengthen the discussion of the gap by emphasizing the emerging need of gamification in teacher education and provide more compelling justifications to why a bibliometric study is necessary for this topic. 

 4. For the Methodology, justify briefly the search string you have utilized for the bibliometric analysis. A sentence may capture the importance of the operators and the identified keywords in the search string. This may be added in Step 3.  

 5. Include figures in the Results section that will support discussion, such as the countries that are at the forefront of the topic and the most productive journals. 

 6. Kindly make the heading more grammatically smooth (i.e. Forefront Countries and Institutions in [Topic], etc.)

 7. For the results on the co-citation network, kindly elaborate how the clusters work together leading to a prolific study of gamification in teacher education. Consider adding a paragraph that views these clusters into a unified construct. 

 8. For the co-occurrence analysis, kindly reflect whether the 7 clusters are relevant to the analysis. Are some of the clusters just interrelated with the other clusters? 

 9. Discussion on periodical progression of themes makes the paper richer, but kindly reflect whether there are only two periods in the emergence of the topic. Analyze further whether there is any significant progress during the 2020s, rather than treating 2025 as a single cluster. 

 10. Kindly emphasize in the discussion the potential future trajectory of this topic, supported with the literatures available. 

 Congratulations!

Are the rationale for, and objectives of, the Systematic Review clearly stated?

Yes

Is the statistical analysis and its interpretation appropriate?

Yes

If this is a Living Systematic Review, is the ‘living’ method appropriate and is the search schedule clearly defined and justified? (‘Living Systematic Review’ or a variation of this term should be included in the title.)

Not applicable

Are sufficient details of the methods and analysis provided to allow replication by others?

Yes

Are the conclusions drawn adequately supported by the results presented in the review?

Yes

Reviewer Expertise:

Mathematics Education, Pre-service Teacher Education

I confirm that I have read this submission and believe that I have an appropriate level of expertise to confirm that it is of an acceptable scientific standard, however I have significant reservations, as outlined above.
